# Feasibility of a theoretically grounded, multicomponent, physiotherapy intervention aiming to promote autonomous motivation to adopt and maintain physical activity in patients with lower-limb osteoarthritis: protocol for a single-arm trial

**DOI:** 10.1186/s40814-023-01274-6

**Published:** 2023-03-31

**Authors:** Matthew Willett, Alison Rushton, Gareth Stephens, Sally Fenton, Sarah Rich, Carolyn Greig, Joan Duda

**Affiliations:** 1 Centre of Precision Rehabilitation for Spinal Pain, University of Birmingham, Birmingham, UK; 2 School of Sport, Exercise and Rehabilitation Sciences, University of Birmingham, Birmingham, UK; 3 School of Physical Therapy, Elborn College, Western University, London, Canada; 4 Research and Development, The Royal Orthopaedic Hospital NHS Foundation Trust, Birmingham, UK; 5 NIHR Birmingham Biomedical Research Centre, University Hospitals Birmingham NHS Foundation Trust and University of Birmingham, Birmingham, UK; 6 MRC-Versus Arthritis Centre for Musculoskeletal Ageing Research, University of Birmingham, Birmingham, UK

**Keywords:** Feasibility, Acceptability, Physical activity, Physiotherapy, Osteoarthritis, Adoption, Maintenance

## Abstract

**Background:**

Lower-limb osteoarthritis (OA) causes high levels of pain and disability in adults over 45 years of age. Adopting and maintaining appropriate levels of physical activity (PA) can help patients with lower-limb OA self-manage their symptoms and reduce the likelihood of developing secondary noncommunicable diseases. However, patients with lower-limb OA are less active than people without musculoskeletal pain. This single-arm feasibility trial seeks to determine the feasibility and acceptability of a complex multicomponent physiotherapy behaviour change intervention that aims to aid patients with lower-limb OA to adopt and maintain optimal levels of PA.

**Methods:**

This trial will be conducted at one site in a National Health Service physiotherapy outpatient setting in the West Midlands of England. Up to thirty-five participants with lower-limb OA will be recruited to receive a physiotherapy intervention of six sessions that aims to optimise their PA levels during phases of behavioural change: adoption, routine formation and maintenance. The intervention is underpinned by self-determination theory (and other motivational frameworks) and seeks to foster a motivationally optimal (empowering) treatment environment and implement behaviour change techniques (BCTs) that target PA behaviours across the three phases of the intervention. Physiotherapists (*n* = 5–6) will receive training in the why and how of developing a more empowering motivational environment and the delivery of the intervention BCTs. Participants will complete patient-reported and performance-based outcome measures at baseline and 3-month (to reflect behavioural adoption) and 6-month (maintenance) post-baseline. Feasibility and acceptability will be primarily assessed through semi-structured interviews (purposively recruiting participants) and focus groups (inviting all physiotherapists and research staff). Further evaluation will include descriptive analysis of recruitment rates, loss of follow-up and intervention fidelity.

**Discussion:**

A novel complex, multicomponent theoretical physiotherapy behaviour change intervention that aims to create a more empowering motivational treatment environment to assist patients with lower-limb OA to adopt and maintain optimal PA levels has been developed. Testing the feasibility and acceptability of the intervention and its associated physiotherapist training and related trial procedures is required to determine whether a full-scale parallel group (1:1) randomised controlled trial to evaluate the interventions effectiveness in clinical practice is indicated.

**Trial registration:**

Trial register: International Standard Randomised Controlled Trial identification number: ISRCTN12002764.

Date of registration: 15 February 2022.

**Supplementary Information:**

The online version contains supplementary material available at 10.1186/s40814-023-01274-6.

## Background

Osteoarthritis (OA) is the leading cause of individual level disability for elderly adults [1] and a significant socioeconomic burden [2] which is anticipated to continue to increase [3] and engulf global healthcare systems within 10 years [4]. Lower-limb (hip and knee) OA is of primary concern, with over 300 million global cases reported, which represents an increase of over 9% from figures detailed in 1990 [5]. In the United Kingdom (UK), approximately one-in-three people over 45 years, equating to 6.6 million individuals, experience lower-limb OA symptoms [1].

Strategies to promote physical activity (PA), including planned exercises and lifestyle activities [6], are fundamental aspects of non-pharmacological interventions to help patients reduce pain and optimise function in several international OA guidelines [1, 7–9]. Physiotherapists are specialists in the assessment and management of musculoskeletal conditions and the primary healthcare provider of PA interventions within the National Health Service (NHS) in the UK [10].However, physiotherapy PA interventions help patients with lower-limb OA to moderate their clinical symptoms only over short time periods (≤ 3-month post-baseline) with symptom recurrence about 6-month post-baseline [11, 12]. Therefore, patients with lower-limb OA may seek further treatment, which places additional strain on the healthcare services. Clinical symptom regression and subsequent re-referral are likely associated with difficulties that patients with lower-limb OA experience when maintaining their prescribed PA behaviours post-discharge [13].

The Medical Research Council (MRC) [14] recommends that behaviour change theory should be utilised when developing complex interventions. Using theory enables specific behavioural determinants (e.g. self-efficacy for PA) to be targeted by the interventions’ treatment techniques [i.e. behaviour change techniques (BCTs)] [15]. Therefore, the interventions active ingredients can be modified over time, leading to potential increased clinical effectiveness on the target behaviour [15]. However, there is a lack of theoretical behaviour change interventions tested on patients with musculoskeletal pain [15].

Researchers postulate that individuals undergo several ‘phases’ of behaviour change when incorporating new behaviours into their lifestyle, with the most important phases for intervention delivery being *adoption/initiation* and *maintenance* [16]. In regard to the promotion of PA by physiotherapists, it is assumed that patients will be *adopting* PA behaviours, while receiving physiotherapy treatment and *maintenance*will be manifested and hopefully ongoing post-discharge. While it is likely that several BCTs are consistently relevant to the behaviour change process overall, others are more pertinent to either the adoption or maintenance of PA phase [17].

Randomised controlled trials (RCTs) are considered the gold standard procedure to evaluate the clinical effectiveness of an intervention. However, due to the complex processes and costs that occur when delivering and evaluating behaviour change interventions, robust scoping work prior to a definitive RCT is essential [18]. Feasibility trials are advocated to test implementation of the interventions’ procedures [19] and trial design [18], thus informing whether a full-scale RCT is warranted [20]. To date, one theoretical physiotherapy behaviour change feasibility trial has been undertaken on patients with lower-limb OA. Hurley et al. (2020) [21] evaluated the feasibility and acceptability of a complex group intervention that aimed to promote self-management behaviours in patients with musculoskeletal pain. Although the intervention was highly acceptable, eligible participants preferred to be managed individually (i.e. one to one). Low recruitment rates and delays in commencing the group intervention led to a full-scale trial being unfeasible to deliver [21]. These issues have also been highlighted in research where patients and physiotherapists did not feel they received, or were unable to deliver, effective personalised treatment within the current time slots employed in the National Health Service (NHS) [22]. Furthermore, physiotherapists most commonly treat patients with lower-limb OA 1 to 1 in clinical practice [23], and this delivery maybe more effective than group classes at aiding patients’ self-management of their levels of pain and function [24].

To address these issues, an individually delivered, complex, multicomponent theoretical behaviour change physiotherapy intervention has been developed. Briefly, the intervention and associated training programme (*Empowering Physio*™) are underpinned by self-determination theory (SDT) which focuses on the underlying reasons for individuals’ behavioural engagement [25]. The overarching aim of the intervention is to create a more motivationally empowering treatment climate to deliver BCTs that target the determinants of PA adoption and maintenance in patients with lower-limb OA.

### Aim

The trial has two primary aims:

1. To investigate the feasibility and acceptability of a complex, multicomponent theoretical behaviour change physiotherapy intervention in the view of patients with lower-limb OA and treating physiotherapists
2. To evaluate the feasibility and acceptability of trial-related procedures to participants and research-related staff

### Objectives

#### Intervention feasibility and acceptability

To determine the feasibility and acceptability of the complex and multicomponent behaviour change intervention to participants and treating physiotherapists

To determine the acceptability of the bespoke *Empowering* *Physio* training programme to treating physiotherapists

To evaluate fidelity of intervention delivery by treating physiotherapists

#### Trial feasibility and acceptability

- To measure the recruitment rates of participants
- To measure the completeness of data collection of performance-based and patient-reported outcome measures at baseline, 3- and 6-month post-baseline
- To determine the feasibility and acceptability of trial-related procedures (recruitment, outcome assessment) to participants and research staff
- To determine the feasibility and acceptability of utilising an accelerometer as the primary outcome in a definitive randomised controlled trial
- To determine the acceptability of the patient-reported and performance-based outcome measures to participants

## Methods

### Ethics

The trial protocol has received a favourable ethical approval from the Health Research Authority via the Integrated Research Application System (IRAS number: 303710). This will ensure that the trial is conducted following best practice ethical guidelines. It will follow guidance from the Research Governance Framework for Health and Social Care and comply with the Data Protection Act 2018. Although clarification will be sought from the research team if a participant withdraws, they may do so without offering one, and their normal care will not be affected in anyway. If a person withdraws, data collected up to the point of withdrawal will be used to inform the analysis unless the participants specifically withdraw consent for this.

### Trial design

**Fig. 1:**
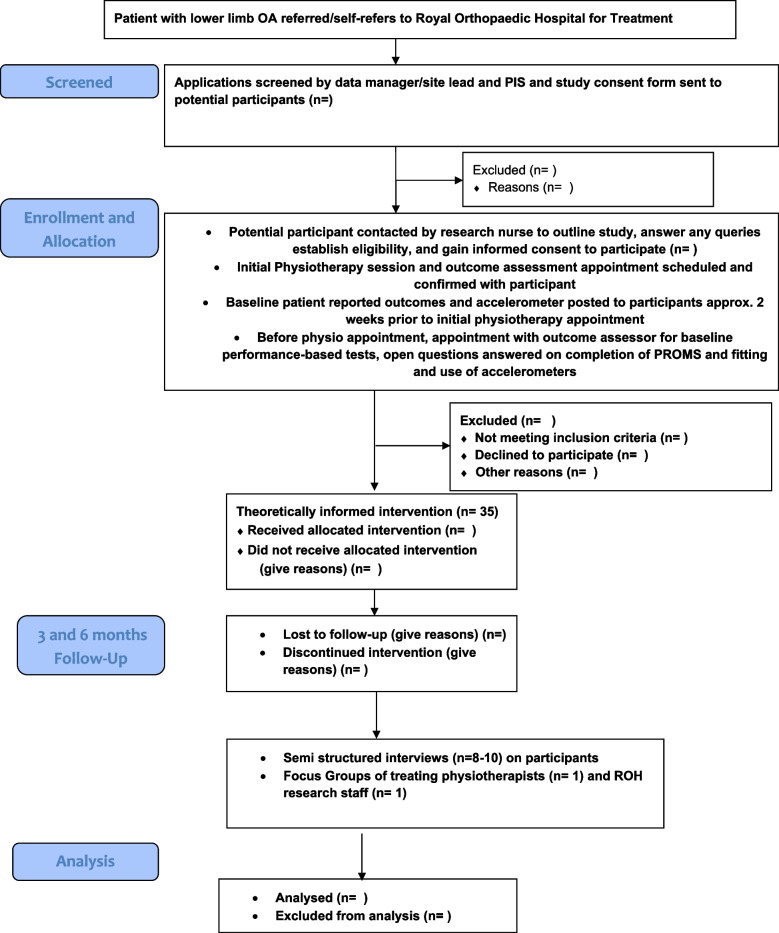
Feasibility trial flow diagram

**Fig. 2:**
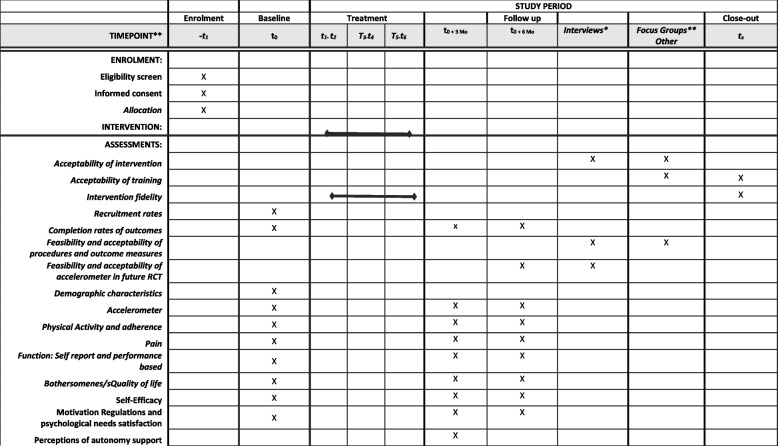
Schedule of enrolment, interventions and assessments

The trial will utilise a single-centre mixed-methods feasibility design including a quantitative prospective, single-arm feasibility trial with a 6-month follow-up [18, 26] and a qualitative component to provide an in-depth examination of the intervention’s feasibility and acceptability [20] in the view of participants, physiotherapists and research staff. Six months are the planned primary end point of the anticipated future definitive RCT. This is based on the generally accepted definition of behavioural maintenance in the literature [27] and large drop off in reported clinical effectiveness on PA behaviours [12] around this time point. This trial is registered with the International Standard Randomised Controlled Trial Number (ISRCTN) database (Trial ID: ISRCTN12002764) and follows Standard Protocol Items Recommendations for Interventional Trials (SPIRIT) guidelines [28]. The trial is reported in accordance with the CONSORT Statement for Pilot and Feasibility studies [29] and the COnsolidated criteria for REporting Qualitative research (COREQ) guidelines for quantitative and qualitative components respectively [30]. Figures 1 and 2 outline the flow of patients with lower-limb OA through the trial and the schedule of enrolment, intervention and assessments, respectively.

### Trial setting and participant eligibility criteria

Patients referred for physiotherapy treatment of their hip or knee OA symptoms at the Royal Orthopaedic Hospital (ROH), Birmingham, will be invited to participate (please see Table 1 for eligibility criteria).

**Table 1:** Eligibility criteria

Inclusion criteria
Diagnosis of unilateral or bilateral lower-limb OA based on the NICE guidelines: [1] males or females ≥ 45 years old as follows:
• Morning joint-related stiffness lasting ≤ 30 min or no morning joint-related stiffness
• Activity-related joint pain
Exclusion criteria
• A further joint-related pathology that could affect the biomechanics of the hip or knee, e.g. rheumatoid arthritis or lumbar nerve root lesion
• Previously had or awaiting knee or hip joint replacement
• Wheelchair dependent
• Unwilling/unable to give informed consent for treatment
• Unable to communicate fluently in English
• Diagnosed with a psychiatric illness (e.g. schizophrenia)
• Diagnosed with an upper motor neuron lesion, e.g. multiple sclerosis
• Unwilling/unable to attend physiotherapy sessions

All intervention procedures will take place in the ROH physiotherapy clinics or virtually via phone or ‘Attend Anywhere’, the secure NHS video call service. Ongoing PA will be undertaken by people with lower-limb OA in their own home or the community.

### Participant identification and consent

#### Feasibility trial

Potential participants will be identified from the physiotherapy waiting lists at the ROH by a member of the research team. If the patient appears to meet the eligibility criteria, they will be sent a participant information sheet (PIS) and trial consent form via post. Each potential participant will be phoned by a research nurse a minimum of 1 week later. Potential participants will be asked if they have reviewed the PIS and if they are interested in trial participation. If they report being interested, the research nurse will confirm eligibility, outline the trial and answer any outstanding questions. Consent will be taken by the research nurse during the phone call and witnessed by another ROH staff member. During the meeting, the research nurse will reiterate that participants are free to withdraw from the trial at any time, and that this will not impact on their current or future healthcare [31].

#### Semi-structured interviews with participants

A purposive sample of patients who undergo the physiotherapy intervention (*n* = 8–10) will be invited to attend individual semi-structured interviews. Participation in the semi-structured interviews will be discussed at the 3-month post-baseline assessment with the research nurse, and consent will be gained in the same manner as for the main trial. If the participant consents to having an interview, the lead researcher (MW) will contact the participant approximately 1 week later. If participants choose not to engage with the interviews, the research nurse will gently probe for any underlying reasons. If the participant chooses to divulge their reasons, these will be anonymously fed back to the lead researcher for data recording and further discussion in study steering meetings. Interviews will be offered either by telephone or skype/zoom depending on the participant’s preference and will be scheduled as close to the end of their treatment sessions as convenient for the participant.

#### Focus groups with Royal Orthopaedic Hospital physiotherapists and research staff

All physiotherapists who deliver intervention (*n* = 6–7) and the ROH trial research staff team will be invited to attend focus groups. The participating ROH physiotherapists and research staff will be approached by the site lead researcher (GS), an advanced physiotherapy practitioner within the ROH. Prior to the focus groups, participants will be given the opportunity to ask any questions, and the PIS will be reviewed in real time. MW will conduct all semi-structured interviews and focus groups and establish informed consent prior to focus groups (intervention physiotherapists and ROH research staff, respectively). MW is experienced in clinical practice, establishing consent (has in-date GCP training), and conducting and analysing qualitative data obtained from semi-structured interviews and focus groups.

#### Multicomponent behaviour change intervention

The complex, multicomponent theoretical behaviour change intervention was developed iteratively through several interconnecting projects including the following:

- A systematic review that identified the most effective BCTs used in physiotherapy interventions to promote PA adherence in patients with lower-limb OA [12, 32]
- Semi-structured interviews with patients with lower-limb OA [17, 33]
- Focus groups with outpatient physiotherapists
- A scoping review and mapping exercise to identify appropriate theories of behaviour change to underpin the intervention delivery and physiotherapy training

The resulting intervention, which is underpinned by self-determination theory (SDT), has the overarching aim of creating a more motivationally empowering treatment climate and to implement particular BCTs to support patients with lower-limb OA to adopt and maintain appropriate individual levels of PA. Briefly, SDT outlines three universal psychological needs (competence, relatedness and autonomy) which influence the quality of an individuals’ motivation (i.e. how self-determined that motivation is) [34]. An individual’s competence relates to their ability to effectively do the behaviour; autonomy refers to whether the engagement is voluntary and the degree of agency that an individual has with regard to the behaviour; relatedness involves feelings of being associated and optimally supported by others in regard to the behaviour [25]. SDT postulates that fulfilment of the 3 psychological needs leads to more autonomous forms of motivation and facilitates behaviour adoption and maintenance. A primarily SDT-based training programme *Empowering Coaching, * [35, 36], which has been adapted for delivery to physiotherapists (i.e. the bespoke *Empowering Physio* training), was selected to guide the training of the trial physiotherapists in regard to the motivational aspects of the intervention delivery. A brief overview of the sequential development of the intervention and associated training programme is outlined in the additonal file 2, Appendix 1 and further detail will be provided in a subsequent paper.

#### Intervention materials and associated behaviour change techniques

Support material has been developed and will be utilised to assist delivery of intervention BCTs. Participants will be given two paper-based workbooks. The first workbook targets facilitating PA adoption by encouraging the patient to reflect on their PA preferences to identify appropriate patient-centred PA goals (‘goal setting’; ‘review behaviour goals’) and includes a weekly activity planner (‘action planner’). Each participant will be given a Yamax pedometer (Tokyo, Japan) to keep that will act as a self-monitoring device (‘self-monitoring of behaviour’; ‘feedback of behaviour’) and as a thanks for partaking in the trial.

Participants will be given the second workbook in session 4. This workbook aids participants to identify necessary modifications to their physical and/or social environment (‘problem-solving’; ‘restructuring of the social environment’) to help develop a PA routine and then maintain these behaviours (‘habit formation’; ‘generalisation of the target behaviour’). A detailed list of local PA services and supports is also included [‘social support (practical)’]. Each workbook is written using needs supportive language to aid participants’ feelings of competence, autonomy and relatedness towards establishing PA as a lifestyle behaviour.

The trial-specific workbooks will be supported by the *Versus Arthritis* information booklets on knee or hip OA which outline general information on OA epidemiology, pathophysiology, management strategies (exercise and medication) and appropriate support services (‘information on health consequences of performing the behaviour’; ‘information on social and environmental consequences’). An outline of the intervention materials, their intended sequencing and associated BCTs is outlined in Table 2.

**Table 2:** Intervention materials delivered across sessions and outline of content with associated theoretical constructs and behaviour change techniques

Session numbers	Intervention material	Intervention content	SDT constructs targeted	Associated behaviour change techniques
**1–2**	Activity Adoption Workbook	Aims of physiotherapy information on benefits of PA Exercises to identify patient-centred PA goals Activity diary	Autonomy Competence	• Goal setting (behaviour and outcome) • Problem-solving • Review behaviour goals (and/or outcome) • Action planning • Self-monitoring of behaviour (and/or outcomes of behaviour)
	Pedometer	Pedometer to self-monitor number of steps	Autonomy Competence	• Self-monitoring of behaviour (and/or outcomes of behaviour) • Feedback on behaviour (and/or outcomes)
	Versus Arthritis Book: Osteoarthritis of the Knee/Hip General Information	Epidemiology of OA Pathophysiology of OA General treatment strategies		• Information on health consequences of performing the behaviour • Information on social and environmental consequences • Modelling/demonstration of behaviour
**3–4**	Routine Formation and Maintenance Booklet	Problem-solving exercises to identify barriers and solutions to physical and social environment as needed to maintain PA Detail of local physical activity resources and support	Competence Autonomy Relatedness	• Problem-solving • Social support (practical) • Habit formation • Generalisation of a target behaviour • Restructuring of the social environment
**Any**	Theraband	Resistance band to enhance or make prescribed exercises more difficult	Autonomy Competence	• Prompts and cues • Habit formation • Adding objects to the environment

Legend: *SDT* Self-determination theory

The use of manual therapy techniques and supportive braces/walking devices will be incorporated into the intervention at the discretion of the treating physiotherapist [1]. Participants will be asked to not seek any additional treatments for their OA symptoms for the duration of the trial and to maintain their standard medications. Any co-interventions will be recorded in the assessment appointments with the research nurses.

#### Intervention delivery

Participants will attend individual intervention sessions (approximately six based on patient preference reflected in previous work related to the intervention development [33] and the standard number of physiotherapy sessions offered by the ROH) with the trained physiotherapists. Physiotherapy sessions will be conducted in person, via phone or ‘Attend Anywhere’ (based on participant preferences). Appointment frequency will depend on the participants’ availability and related PA goals and the physiotherapists’ clinical schedules. The ROH’s standard physiotherapy sessions are 45 min for an initial appointments and 30 min for a follow-up session.

#### Physiotherapist training

The bespoke *Empowering Physio*™ training programme aims to enhance physiotherapists’ understanding of differences in the quality of patient motivation (and implications) and awareness of the treatment climate they create and how it can influence patients’ psychological needs of competence, autonomy, relatedness and their subsequent autonomous motivation to adopt and maintain their PA goals. The training will involve presentations, and interactive exercises to convey why, when and how physiotherapists interact and provide feedback with patients during their treatment sessions may influence participants’ levels of controlled and autonomous motivation to engage in PA. Delivery of the BCTs across PA adoption, routine formation and/or maintenance will be addressed including their theoretical background. The physiotherapists will receive printed and electronic versions of the *Empowering Physio*™ training workbook, a handbook with BCT definitions and clinical examples and key research articles relevant the intervention.

Intervention physiotherapists (*n* = 6–7) will be trained by two members of the research team, Professor Joan Duda (JD), the founder of the *Empowering Coaching* programme who possesses a PhD in psychology and is an internationally renowned expert in motivational processes, and MW, whose PhD has focused on the development of the intervention. Training will include two 3-h, in-person group training sessions conducted on consecutive weeks and a follow-up 2-h ‘top-up’ session approximately 1 month later.

#### Trial outcomes and success criteria

Once data analyses are complete, the overall trial’s success (i.e. trial-related procedures and intervention feasibility and acceptability) will be determined by evaluating the results against a priori established criteria for success (Table 3). The criteria were established by the Trial Management Group, which includes experts in musculoskeletal physiotherapy, ROH physiotherapists and researchers with expertise in trial methodology and behaviour change supported by contemporary literature in contemporary feasibility trial methodology [37]. Each criterion will be evaluated against guidance outlined by Thabane et al. (2010) [38] to determine if progression to a definitive trial is recommended. Possible outcomes include the following:

- Stop: A definitive trial is not feasible.
- Continue with modification: Modification of the protocol is required to make definitive trial feasible.
- Continue without modification: Modification of the protocol is not required to make definitive trial feasible but requires close monitoring.
- Continue without modifications: A definitive trial appears feasible.

**Table 3:** Trial outcomes and success criteria

Aim 1: Intervention feasibility and acceptability		
**Objective**	**Method of assessment**	**Success criteria**
**Objective 1.1:** To determine the feasibility and acceptability of the complex and multicomponent behaviour change intervention to participants and treating physiotherapists (P)	• Participants: Semi-structured interviews •Physiotherapists: Focus group	**Continue without modifications**: Thematic analysis identifies that trial participants and treating physiotherapists found the intervention satisfactory (i.e. it was feasible and acceptable) in semi-structured interviews and focus groups respectively **Stop**: Thematic analysis suggests that trial participants and treating physiotherapists did not find the intervention satisfactory (i.e. it was unfeasible and/or unacceptable)
**Objective 1.2:** To determine the acceptability of the bespoke Empowering Coaching training programme (Empowering Physio) to treating physiotherapists (P)	• Focus group	**Continue without modifications**: Thematic analysis identifies that intervention physiotherapists perceived that the EC training programme developed their understanding and enactment of how to create an empowering treatment climate **Stop**: Thematic analysis suggests that intervention physiotherapists perceived that the EC training programme was insufficient to develop their understanding or enactment of how to create an empowering treatment climate
**Objective 1.3**: To evaluate fidelity of intervention delivery by treating physiotherapists (S)	• Two members of the research team will assess transcripts of one intervention session from the adoption, routine formation and maintenance phases from first and third participants for the following: 1) Intervention content by coding of BCTs 2) Treatment climate using the ISPACOT [39]	• Essential pre-specified BCTs are delivered with at least mean ‘moderate’ levels of fidelity (≥ 50%) [40] • Mean score of ≥ 4/7 on Likert scale when using ISPACOT to evaluate treatment climate [41]
**Aim 2: Trial feasibility and acceptability**		
**Objective**	**Method of assessment**	**Success criteria**
**Objective 2.1**: To measure the recruitment rates of participants (P)	• Recording number (*n*) of participants who were sent a participant information sheet, number who agreed to participate and number recruited per month	**Continue without modification** • Target of *n* = 35 participants recruited in 9 months (approx. 4/month) • Conversion rate of ≥ 30% (number sent PIS/ number who agreed to participate) **Stop** • Target of *n* = 35 participants recruited takes > 12 months (less than 3/month) • Conversion rate of ≤ 15% (number sent PIS/ number who agreed to participate)
**Objective 2.2**: To measure the completeness of data collection of performance-based and patient-reported outcome measures at baseline, 3- and 6-month post-baseline (P)	• Record number of PROMs returned to trial team and number of participants who completed performance-based outcomes during virtual outcome assessments at each timepoint	**Continue without modification** • ≤ 80% PROM and performance-based data collected at 3-month post-baseline • ≤ 70% PROM and performance-based data collected at 6-month post-baseline **Stop** • ≤ 50% PROM and performance-based data collected at 3-month post-baseline • ≤ 50% PROM and performance-based data collected at 6-month post-baseline
**Objective 2.3**: To determine the feasibility and acceptability of trial-related procedures (recruitment, outcome assessment) to participants and ROH research staff ((P)	• Participants: Semi-structured interviews • ROH research staff: Focus groups	**Continue with modification** • Thematic analysis identifies that trial participants and ROH research staff are satisfied (i.e. feasible and acceptable) with trial procedures (e.g. recruitment, outcome assessment) in semi-structured interviews and focus groups respectively **Stop** • Thematic analysis identifies that trial participants and ROH research staff are not satisfied (i.e. they do not perceive it is feasible and/or acceptable) with trial procedures (e.g. recruitment, outcome assessment) in semi-structured interviews and focus groups respectively
**Objective 2.4**: To determine the feasibility and acceptability of utilising an accelerometer as the primary outcome in a definitive randomised controlled trial (S)	• % of accelerometers returned to research staff with valid data • Semi-structured interviews	• ≤ 70% of accelerometer data collected at 6-month post-baseline • Accelerometer data will be considered valid if it has been recorded for ≥ 10 h [42, 43] for ≥ 4/7 days, with at least one falling on a weekend [44–46] • Participants state that they believe that accelerometer is feasible and acceptable to use as the primary outcome measure in future definitive trial
**Objective 2.5**: To determine the acceptability of the patient-reported and performance-based outcome measures to participants ((S)	• Semi-structured interviews	• Participants report that they found the patient-reported and performance-based outcomes feasible and acceptable, and the time taken to complete was satisfactory for use in a definitive randomised controlled trial

Legend: *ISPACOT* Interpersonal support in physical activity consultations observational, *P* Primary feasibility outcome related to progression to definitive trial, *S* Secondary feasibility outcome

Once it has been established if each criterion has been met, further consultation with patient and public involvement (PPI) at Trial Steering Group meetings will be utilised to determine if and what modifications the intervention requires to progress to the full-scale RCT (e.g. adaptions to intervention to enable delivery in alternative NHS physiotherapy departments).

### Methods of data collection

#### Patient-reported and performance-based outcomes during trial

Participants will complete outcome assessment at baseline, 3-month post-baseline and 6-month post-baseline. Baseline outcome assessment appointments will be scheduled immediately prior to participants’ initial physiotherapy session. Outcome assessments are anticipated to be conducted virtually in the participants’ own homes via phone or ‘Attend Anywhere’. However, if participants do not feel confident about completing their performance-based outcomes at home, they will be offered a choice of having their baseline assessment done in person. Outcomes to be assessed include the use of an accelerometer to measure daily PA, patient-reported outcome measures (PROMs) and performance-based outcomes. Accelerometers and PROMs will be posted to participants’ home addresses 1 week prior to outcome assessment appointments. A 150-cm tape measure will be included for standardisation of one of the performance-based tests (figure-of-8 walking test: Table 4) [47]. Participants will be asked to complete PROMs prior to assessment appointments so the session can be used to collect performance-based outcomes and answer any questions. A stamped, addressed envelope will be included for return of accelerometers (including simple fitting instructions and a belt to secure the accelerometer) and PROMs to the ROH. If accelerometers and PROMs are not returned within 2 weeks of their assessment appointment, participants will be contacted via text/telephone and asked to send them back at their earliest convenience.

**Table 4:** Planned participant secondary outcome measures for randomised controlled trial

Outcome domain	Instrument	Administered at	Rationale
Physical activity	Short-Form (7 questions) International Physical Activity Questionnaire [48]	Baseline, 3 months, 6 months	Primary aim of physiotherapy intervention. Has demonstrated reliability and validity [48]
Adherence to PA/exercise recommendations	Section B of the Exercise Adherence Rating Scale (6 items) [49]	Baseline, 3 months, 6-month post-baseline	Primary aim of the physiotherapy intervention. Has demonstrated reliability [49] and validity [50]
Pain	11-point Numerical Pain Rating Scale [51]	Baseline, 3 months, 6 months	Suggested to include as a core outcome domain in international guidelines [51]. Shown excellent reliability [52] and suggested as more reliable than a visual analogue scale in patients who are older or less literate patients and more useful for demonstrating change [53]
Function: Self-report	Short-Form Hip Disability and Osteoarthritis Outcome Score (SF-HOOS)/Short-From Knee Injury and Osteoarthritis Outcome Score (SF-KOOS) [51]	Baseline, 3 months, 6 months	Suggested to include as a core outcome domain in international guidelines [51]. Have shown good levels of reliability [54, 55]
Function: Performance based	Five times sit-stand test	Baseline, 3 months, 6-month post-baseline	Direct, observable measure of physical function. Has demonstrated excellent validity and reliability when tested in home setting [56]
	Figure-of-8 walking test	Baseline, 3 months, 6 months	Direct, observable measure of physical function. Has demonstrated validity and reliability when tested at home [47]
Bothersomeness	Bothersomeness Index: 5-point Likert scale	Baseline, 3 months, 6 months	Outlined as important outcome domain by public involvement representative
Quality of life	EuroQol 5-domain instrument (EQ-5D) [51]	Baseline, 3 months, 6 months	A domain recommended by international guidelines. Has shown good reliability and validity [57]
Self-efficacy for physical activity/exercise	Self-Efficacy for Exercise Scale 9 items [58]	Baseline, 3 months, 6 months	Outlined as an important influence on behaviour change in preliminary work. Measures several aspects of self-efficacy including perceived barriers to exercise [59]. Valid and reliable [58]
Motivation regulations to participate in physical activity/exercise	Behavioural Regulation in Exercise Questionnaire-3 24 items [60, 61]	Baseline, 3 months, 6 months	Motivation was outlined as important influence on behaviour change in preliminary work. Grounded in SDT. Important stage of intervention process model. Valid [60, 62] and reliability [63]
Psychological needs satisfaction	A modified version of the Psychological Need Satisfaction in Exercise Scale [64, 65]	Baseline, 3 months, 6 months	Grounded in SDT. Important stage of intervention process model. Valid and reliable [64, 65]
Social psychological environment/perceptions of autonomy support	Health Care Climate Questionnaire 15 items [66]	3-month post-baseline	Assesses the participants perceptions of the motivational social climate provided in physiotherapy sessions. Grounded in SDT. Important stage of intervention process model. Identified as an important influence on behaviour change in preliminary work. Reliable [66]

The accelerometer will be fitted over the right anterior superior iliac spine [45, 46, 67]. Following a standardised and widely used protocol [68], participants will be asked to wear accelerometers for 7 consecutive days [43, 69–71] with removal for sleep or when in water (e.g. swimming or shower) [45, 46, 71, 72]. The patient-reported and performance-based outcomes to be assessed, including their rationale, are detailed in Table 4.

#### Collection of qualitative data

As the main aim of the qualitative component of the trial is to gain in-depth perspectives on the feasibility and acceptability of the trial intervention and associated processes, the epistemological stance is based on phenomenology [73]. Questions will initially be asked in an open manner with clarification sought if key points are identified. Participants, physiotherapists.and research staff will be invited to introduce new topics and/or issues as the discussions progress. The respective topic guides have been developed a priori with questions determined through collaboration of the trial management team which includes clinical physiotherapists, researchers with expertise in qualitative research methods and behaviour change and PPI [74].

For individuals attending semi-structured interviews and focus groups, demographic detail will be recorded to enable description of participants, intervention physiotherapists and ROH research staff, respectively.

#### Patient and public involvement

All patient-facing documents were designed in collaboration with a lay person (ED) with extensive experience in public involvement and a lifetime’s work in effective communication. ED is a central member of the Trial Management and Steering Groups. They have reviewed and offered extensive feedback on all patient-facing documents including the patient information sheets, consent forms, semi-structured interview forms, intervention content, trial protocol, ethics application and PROMs.

#### Sample size

Although a formal calculation was not completed, total sample sizes of between *n* = 24 to 50 are recommended in feasibility trials and a sample of 30 [18] to 35 [75] considered sufficient to ensure normal distribution of participants. An audit of the ROH physiotherapy waiting lists suggested that approximately 65 patients with musculoskeletal pain are referred to the ROH each week, and that approximately 5% would be eligible to participate in the trial (i.e. three per week). Based on previous trials conducted at the ROH, approximately one in three of those approached consents to participate. Therefore, based on trial resources and timelines, recruitment of a target sample of up to 35 participants over a 9-month period is estimated.

### Data analysis

#### Quantitative

The primary quantitative data analysis will be descriptive [19, 31]. A CONSORT diagram will outline the number of participants who were identified, recruited, commenced and finished treatment, and the recruitment rate (number/month and number approached/number recruited) will be reported as a percentage [29]. Where possible, reasons for refusal and dropout during the intervention will be recorded and reported. A Shapiro–Wilk test will test the normality of continuous data (e.g. PROM and accelerometer data), and the mean and 95% confidence intervals will be reported for each. The number of non-completed/partially completed PROMs, performance measures and accelerometer data at each timepoint will be expressed as a percentage. Partially completed PROM and accelerometer data will not be included in the data analysis, but potential reasons, including practicality, will be discussed in the interviews.

#### Intervention fidelity

Fidelity assessment of delivery of the intervention will include evaluation of two aspects: what was delivered (e.g. the BCTs) and how these BCTs were delivered/overall exchanges between the physiotherapist and patient (i.e. the physiotherapy treatment motivational climate) in regard to promoting the patient’s feelings of competence, autonomy and relatedness. Physiotherapists will be asked to audio record one session from each of the adoption, routine formation and maintenance phases from their first and third participants. The specific sessions to be recorded in each intervention phase will be randomised. The audio recording will be transcribed verbatim.

#### What was delivered: evaluation of behaviour change techniques delivered

- Two members of the research team will code all transcripts to identify the BCTs delivered. A coding manual will be provided to coders to optimise consistency. It will include intervention BCTs definition as per the V1 taxonomy [76] and examples of how the technique may be delivered or described in the intervention. BCTs will only be coded once per session and will be written as either (1) fully delivered or (2) partially delivered [77]. BCTs that were present but not part of the specific protocol will also be noted [78]. Coders will have completed online training for BCT identification using the V1 taxonomy and have experience coding BCTs from intervention manuscripts [12] and transcribed interviews with high levels of reliability. The median and interquartile range of BCTs per session in the adoption, routine formation and maintenance phases will be reported respectively. The number of times each BCT was fully delivered compared to when it was outlined in the treatment manual will be averaged across physiotherapists and calculated as a percentage. Overall fidelity will be categorised as *high* if ≥ 80% of essential BCTs are present, *moderate* if 50–79% are present, and *low*if 0–49% are present [40, 78].

#### How was it delivered: evaluation of the treatment motivational climate

Two researchers will use the Interpersonal Support in Physical Activity Consultations Observational Tool (ISPACOT) [39], a 21-item tool that is grounded in SDT, which will be used to evaluate the treatment climate of the physiotherapy sessions. The tool assesses four aspects of the motivational climate created as follows: autonomy support, involvement, structure and interpersonal control and has demonstrated moderate to high levels of inter-rater reliability when assessments are carried out by trained users [39]. A 7-point Likert scale, ranging from 1 ‘not at all true’ to 7 ‘very true’, will be used to determine the degree of need support. The mean Likert scores will be calculated for all physiotherapists and reported for the adoption, routine formation and maintenance phases, respectively. In line with previous research, scores of ≥ 4/7 will be considered the minimum required to deliver a need supportive treatment climate [41, 79].

#### Qualitative data analysis

The interviews and focus groups will be audio recorded and transcribed verbatim. Interviews and focus groups will not be repeated. However, transcripts will be returned to participants/physiotherapists/research staff for member checking and any supplementary comments which are highlighted will be presented to the Trial Steering Group, noted and integrated with the main analysis [80]. MW will take supplementary field notes during the interview process to enable triangulation during data analysis.

All transcripts will be analysed following a six-step deductive method as outlined in Braun and Clarke (2006) [81]. To analyse the semi-structured interviews, two researchers will code the first transcript together to establish a preliminary framework and identify initial themes using NVivo 11 software (QSR International, Melbourne, Australia). A second transcript will be coded independently by the same two researchers with in-depth meetings planned to test the initial framework. The remaining transcripts will be coded by both researchers, with codes and emerging themes clarified through researcher meeting (approximately every two–three transcripts). Focus groups will be undergo the same analysis by two researchers. Iterative results from semi-structured interviews and focus groups, supported by direct quotations, will be presented to the research team and at Trial Steering Group meetings for feedback and challenge.

#### Additional information

Further trial information, including data storage, monitoring of adverse events and intended dissemination of findings, can be viewed in the additional file 2, Appendix 2.

## Discussion

Lower-limb OA is a prevalent and painful condition that impacts the quality of life in adults ≥ 45 years old. International guidelines support optimising PA to help manage clinical symptoms associated with OA. However, people with lower-limb OA are generally less active than other people of a similar age. Optimising PA levels requires people to alter their behaviours, and this is thought to be most easily accomplished if the treatment incorporates behaviour change theory. Theoretical interventions have demonstrated significant improvements in PA levels in several populations with secondary noncommunicable diseases. Therefore, the main aim of this trial is to determine the acceptability and feasibility of delivery of a complex, multicomponent theoretical physiotherapy behaviour change intervention that aims to optimise adoption and maintenance of prescribed PA in patients with lower-limb OA.

The behaviour change intervention has been developed targeting the specific behavioural determinants of PA adoption and maintenance. It has been developed sequentially over a series of projects including a systematic review and a qualitative project incorporating the views of patients with lower-limb OA and heavily influenced from feedback from PPI.

Key methodological considerations were discussed extensively within the SSC at the trial planning stage. These included the primary data collection end point and choice of a single-arm or randomised parallel two-arm trial design.

Within the behaviour change literature, the concept of behavioural ‘maintenance’ suffers from considerable heterogeneity [16]. However, the most utilised definition in empirical studies is that proposed in the transtheoretical model, which suggests behavioural maintenance occurs after approximately 6 months of behaviour change practice (i.e. when a behaviour change intervention commences) [82]. Although this definition is based on addictive behaviours, it has also been utilised in previous systematic reviews examining PA in healthy young and middle-aged [83] and inactive adult populations [84].

Interestingly, the effectiveness of physiotherapy delivered BCTs on PA behaviours (as measured by effectiveness ratios) was lowest at 6-month post-baseline compared to other time points in our systematic review [12]. Furthermore, there appeared to be a drop off in the amount of available data for analysis at this timepoint. Therefore, a 6-month post-baseline was chosen as the primary end point for our feasibility study. However, if a definitive RCT is indicated once analysis is complete, it is anticipated that the interventions effectiveness will be measured at further time points post-baseline (e.g. 9 and/or 12 months) to capture this data for further analysis.

There have been an extensive number of RCTs conducted on patients with lower-limb OA, and methodological aspects such as randomisation have already been established. As information on these features exists, and STAPLO feasibility trial does not aim to test the interventions effectiveness, adding a parallel arm was considered unresourceful and potentially unethical. To this end, a single-arm study with a sample size great enough to provide a normal distribution of patients was planned [85].

This trial design should enable purposive sampling to gain a wide range of opinions on the feasibility and acceptability of the intervention from participants, intervention physiotherapists and research staff. Furthermore, study research nurses were not informed of the trials’ overall design (single arm versus parallel arms) or primary aims during training. Therefore, they are considered blind assessors in the STAPLO trial. The results of several large scales, low risk-of-bias RCTs, from those identified in our systematic review, [12, 86] and conducted locally, [87] will be used to inform methodological decisions, such as a sample size calculation, if a full-scale definitive trial is indicated.

The proposed intervention protocol has been concept tested against physiotherapists working clinically and further refined to encompass theories of behaviour change. Feasibility examination is required prior to its effectiveness being determined.

## Supplementary Information

**Additional file 1. **The STaying Active with Physiotherapy in patients with Lower-limb Osteoarthritis (STAPLO): Feasibility Trial – Consent Form. The STaying Active with Physiotherapy in patients with Lower-limb Osteoarthritis (STAPLO): Feasibility Trial – Interview Consent Form.  Physiotherapist focus group consent form. Research staff focus group consent form.

**Additional file 2:** **Appendix 1**. Overview of intervention development. **Appendix 2.** Additional Trial Information. Table 1: Adverse Event Definitions. 

## Data Availability

The datasets used and/or analysed during the current trial will be available from the corresponding author on reasonable request.

## Abbreviations

OA: Osteoarthritis
PA: Physical activity
BCT: Behaviour change technique
UK: United Kingdom
NHS: National Health Service
MRC: Medical Research Council
SDT: Self-determination theory
CONSORT: ISRCTN: International Standard Randomised Controlled Trial Number
SPRIT: Standard Protocol Items Recommendations for Interventional Trials
COREQ: COnsolidated criteria for REporting Qualitative research
ROH: Royal Orthopaedic Hospital Birmingham
PIS: Participant information sheet
PROM: Patient-reported outcome measure
EC: Empowerment coaching
V1: Version 1
ISPACOT: Interpersonal Support in Physical Activity Consultations Observational Tool
