# Supplementary material for: Feasibility of a theoretically grounded, multicomponent, physiotherapy intervention aiming to promote autonomous motivation to adopt and maintain physical activity in patients with lower-limb osteoarthritis: protocol for a single-arm trial
Source: Pilot Feasibility Stud. 2023 Mar 31;9:54. doi: 10.1186/s40814-023-01274-6 (PMC10064730; doi:10.1186/s40814-023-01274-6)
Supplement: Supplementary file 1 — Additional file 1. The STaying Active with Physiotherapy in patients with Lower-limb Osteoarthritis (STAPLO): Feasibility Trial – Consent Form. The STaying Active with Physiotherapy in patients with Lower-limb Osteoarthritis (STAPLO): Feasibility Trial – Interview Consent Form. Physiotherapist focus group consent form. Research staff focus group consent form. [file 40814_2023_1274_MOESM1_ESM.docx]

## The STaying Active with Physiotherapy in patients with Lower-limb Osteoarthritis (STAPLO): Feasibility Trial – Consent Form

**Please initial each box**

1. I have read and understand the Patient information sheet V01 dated xxxxx,

for the above study. I have had the opportunity to consider the information,

to ask questions and have had these answered satisfactorily.

1. I understand that my participation is voluntary and that I am free to withdraw at any time, without giving any reason, without my medical care or legal rights being affected.
2. I understand that I will be attending the physiotherapy treatment programme which aims to help me be as active as possible. The sessions will take place at the Royal Orthopaedic Hospital or via phone or the ‘Attend Anywhere’ virtual platform
3. I understand that all data will be anonymised and securely stored at the University of Birmingham. My contact details or any identifiable data will be stored for the duration of the study and then destroyed. My data will be stored for 10 years
4. I agree for some of my treatment sessions to be audio recorded and what I say transcribed unidentifiably.
5. I agree for the University of Birmingham trial researchers, NHS sites and regulatory bodies to have access to the anonymised data
6. I agree to take part in the above study
7. We would be interested in hearing your feedback about the physiotherapy course. If you interested in attending an interview and giving your opinion about the physio course, please initial the box alongside this statement

___________ _____

Name of Participant Date Signature

___________ _____

Name of staff member taking consent Date Signature

**For witnessed verbal consent:** I witnessed accurate reading of the consent form to the patient, who could ask any questions and was happy with the responses.

___________ _____

Name of staff who witnessed consent Date Signature

When completed, 1 for participant; 1 for Uni of Birmingham; 1 for the site file

## The STaying Active with Physiotherapy in patients with Lower-limb Osteoarthritis (STAPLO): Feasibility Trial – Interview Consent Form Please initial each box

1. I have read and understand the Interview Participant Information Sheet V01 dated xxxxx,

for the above study. I have had the opportunity to consider the information,

to ask questions and have had these answered satisfactorily.

1. I understand that my participation is voluntary and that I am free to withdraw at any time, without giving any reason, without my medical care or legal rights being affected.
2. I understand that all data collected during the interview will be anonymised and securely stored at the University of Birmingham. My contact details or any identifiable data will be stored for the duration of the study and then destroyed. The data analysis of what I say will be stored for 10 years.
3. I understand that, if I withdraw from the study two weeks or more after interview, my transcribed data will be used in the analysis
4. I agree for my interview to be audio recorded and what I say transcribed unidentifiably.
5. I agree for the University of Birmingham trial researchers, NHS sites, and regulatory bodies to have access to the anonymised data
6. I understand that publications may include anonymised quotations of what I say
7. I agree to take part in the interview
8. I understand that publications may include quotations but that I will not be identifiable from them.
9. If you interested in receiving a copy of the results, please write your email here:

________________________________________

___________ _____

Name of Participant Date Signature

___________ _____

Name of staff member taking consent Date Signature

**For witnessed verbal consent:** I witnessed accurate reading of the consent form to the patient, who could ask any questions and was happy with the responses.

___________ _____

Name of staff who witnessed consent Date Signature

When completed, 1 for participant; 1 for Uni of Birmingham; 1 for the site file

**PHYSIOTHERAPIST FOCUS GROUP CONSENT FORM**

**Please initial box**

1. I confirm that I have read and understand the Participant information sheet V1: Focus groups dated X/XX/XX, for the above study. I have had the opportunity to consider the information and to ask questions and have had these answered satisfactorily.
2. I understand that my participation is voluntary and that I am free to withdraw at any time, without giving any reason, without my occupation or legal rights being affected.
3. I understand that all data will be confidential and securely stored in the REDCap computer system at the University of Birmingham. My contact details or any identifiable data will be stored for the duration of the study and then destroyed. The transcripts and associated data analysis will be stored for 10 years
4. I understand that if I withdraw from the study more than two weeks after the focus group has been conducted, the transcribed data of what I say in the focus group will be used in the analysis

I agree for the focus group discussions to be audio recorded and my pseudonomised data transcribed by the ‘transcription centre’ which is an external provider recommended by the University of Birmingham ([www.transcriptioncentre.co.uk](https://mail.bham.ac.uk/owa/redir.aspx?C=0dmIsYXrhdPF9YedM03KJuHH1EdhHsmpSqCTf3_sqClZIF-WaMXVCA..&URL=http%3a%2f%2fwww.transcriptioncentre.co.uk))

1. I agree for the Sponsor, NHS sites, and regulatory bodies to have access to the data

.

1. I agree to take part in the above study
2. I understand that publications may include quotations but that I will not be identifiable from them.

________________________ ________________ ____________________

Name of Participant Date Signature

_________________________ ________________ ____________________

Researcher Date Signature

When completed, 1 for participant; 1 for researcher; 1 for the site file.

**RESEARCH STAFF FOCUS GROUP CONSENT FORM**

**Please initial box**

1. I confirm that I have read and understand the Participant information sheet V1: Focus groups dated X/XX/XX, for the above study. I have had the opportunity to consider the information and to ask questions and have had these answered satisfactorily.
2. I understand that my participation is voluntary and that I am free to withdraw at any time, without giving any reason, without my occupation or legal rights being affected.
3. I understand that all data will be confidential and securely stored in the REDCap computer system at the University of Birmingham. My contact details or any identifiable data will be stored for the duration of the study and then destroyed. The transcripts and associated data analysis will be stored for 10 years
4. I understand that if I withdraw from the study more than two weeks after the focus group has been conducted, the transcribed data of what I say in the focus group will be used in the analysis

I agree for the focus group discussions to be audio recorded and my pseudonomised data transcribed by the ‘transcription centre’ which is an external provider recommended by the University of Birmingham ([www.transcriptioncentre.co.uk](https://mail.bham.ac.uk/owa/redir.aspx?C=0dmIsYXrhdPF9YedM03KJuHH1EdhHsmpSqCTf3_sqClZIF-WaMXVCA..&URL=http%3a%2f%2fwww.transcriptioncentre.co.uk))

1. I agree for the Sponsor, NHS sites, and regulatory bodies to have access to the data

.

1. I agree to take part in the above study
2. I understand that publications may include quotations but that I will not be identifiable from them.

________________________ ________________ ____________________

Name of Participant Date Signature

_________________________ ________________ ____________________

Researcher Date Signature

When completed, 1 for participant; 1 for researcher; 1 for the site file.
