# Supplementary material for: Feasibility of a theoretically grounded, multicomponent, physiotherapy intervention aiming to promote autonomous motivation to adopt and maintain physical activity in patients with lower-limb osteoarthritis: protocol for a single-arm trial
Source: Pilot Feasibility Stud. 2023 Mar 31;9:54. doi: 10.1186/s40814-023-01274-6 (PMC10064730; doi:10.1186/s40814-023-01274-6)
Supplement: Supplementary file 2 — Additional file 2: Appendix 1. Overview of intervention development. Appendix 2. Additional Trial Information. Table 1: Adverse Event Definitions. [file 40814_2023_1274_MOESM2_ESM.docx]

**Additional File 1 for Feasibility of a theoretically grounded, multicomponent, physiotherapy intervention aiming to promote autonomous motivation to adopt and maintain physical activity in patients with lower-limb osteoarthritis: Protocol for a single-arm trial**

**Appendix 1:**

**Overview of intervention development**

The complex, multi-component theoretical behaviour change intervention has been developed sequentially as outlined below.

**Systematic review**

A systematic review evaluated the effectiveness of BCTs within physiotherapy interventions aimed at optimising adherence to PA in patients with lower-limb OA.^1 2^ Most BCTs demonstrated modest effects (measured by likelihood ratios) at optimising PA adherence. Findings also revealed specific techniques (i.e., ‘Action planning’ and ‘Feedback on behaviour’) to be most effective over the short-term (≤ three months post baseline) while others (i.e., ‘Problem solving’ and ‘Review behaviour goals’) were more effective over the longer-term (≥ six months). Some behaviour change techniques (i.e., ‘Behavioural contract’ and ‘Non-specific reward’) were potentially effective across all time points. Overall, BCTs were least effective at promoting PA adherence at six months post-baseline before a slight recovery at 12 months post-baseline.^2^

**Qualitative Studies**

**Interviews with Patients**

Semi-structured interviews^3 4^ examined the views of patients with lower-limb OA regarding the barriers and facilitators to adherence to physiotherapist prescribed PA during treatment (adoption) and post-discharge (maintenance). The results revealed that the promotion of a PA routine within a positive, supportive, patient-centred physiotherapy environment was integral to behavioural adoption and the facilitation of confidence and motivation to initially engage with prescribed PA when outside the clinic. Modifying the patient PA environment to ensure it is positive and supportive and ongoing access to appropriate resources deemed important to facilitate PA maintenance post-discharge. Pulling from these findings, a theoretically informed physiotherapy intervention which incorporated 26 BCTs was developed.

**Focus Groups of physiotherapists**

The proposed intervention was presented to physiotherapists to assess their perspectives on its acceptability and feasibility for implementation in clinical practice. Although the physiotherapists found the intervention highly acceptable and did not suggest any alteration of intervention BCTs, they believed that they required further training in behaviour change theory, especially in strategies for creating a more supportive motivational treatment environment and how to optimally deliver the BCTs.

**Selection of theory and choice of training programme**

Theories of behaviour change were reviewed to provide a conceptual framework to support training of physiotherapists in intervention delivery. Self-determination Theory (SDT),^5 6^ a prominent theory of behaviour change which focuses on an individuals’ underlying reasons (i.e motivations) for behavioural engagement, was chosen as an appropriate theory to underpin the intervention.^7^ The resulting intervention has the overarching aim of creating a more motivationally empowering treatment climate and to implement particular behaviour change techniques (BCTs) to support patients with lower-limb OA to adopt and maintain appropriate individual levels of PA.

The *Empowering Coaching* programme,^8 9^ which is grounded in SDT, was adapted for delivery for physiotherapists (*Empowering Physio*™). The overarching aim of the bespoke training programme is to enhance physiotherapists’ understanding of differences in the quality of patient motivation (and implications), and awareness of the treatment climate they create and how it can influence patients’ psychological needs of competence, autonomy, and relatedness their subsequent more autonomous motivation to adopt and maintain their PA goals.

**References**

1. Willett M, Duda J, Gautrey C, et al. Effectiveness of behavioural change techniques in physiotherapy interventions to promote physical activity adherence in patients with hip and knee osteoarthritis: a systematic review protocol. *BMJ Open* 2017;7(6):e015833. doi: 10.1136/bmjopen-2017-015833 [published Online First: 2017/07/02]

2. Willett M, Duda J, Fenton S, et al. Effectiveness of behaviour change techniques in physiotherapy interventions to promote physical activity adherence in lower limb osteoarthritis patients: A systematic review. *PLoS One* 2019;14(7):e0219482. doi: 10.1371/journal.pone.0219482 [published Online First: 2019/07/11]

3. Willett MJ, Greig C, Rogers D, et al. Barriers and facilitators to recommended physical activity in lower-limb osteoarthritis: protocol for a qualitative study exploring patients and physiotherapist perspectives using the theoretical domains framework and behaviour change taxonomy. *BMJ Open* 2019;9:e029199. doi: 10.1136/bmjopen-2019-029199 doi: 10.1136/

4. Willett M, Greig C, Fenton S, et al. Utilising the perspectives of patients with lower-limb osteoarthritis on prescribed physical activity to develop a theoretically informed physiotherapy intervention. *BMC musculoskeletal disorders* 2021;22:155. doi: 10.1186/s12891-021-04036-8

5. Deci EL, Ryan RM. Facilitating optimal motivation and psyhcological well-being across life's domains. *Canadian Psychology* 2008;49(1):14-23.

6. Deci EL, Ryan RM. The “what” and “why” of goal pursuits: Human needs and the self-determination of behaviour. *Psychological Inquir* 2000;11(4):227-68.

7. Deci EL, Ryan RM. Intrinsic motivation and self-determination in human behavior. New York: Plenum Press. 1985

8. Duda JL. The conceptual and empirical foundations of Empowering Coaching™: Setting the stage for the PAPA project. *International Journal of Sport and Exercise Psychology* 2013;11(4):311-18. doi: 10.1080/1612197x.2013.839414

9. Duda JL, Appleton PR. Empowering and Disempowering Coaching Climates: Conceptualization, Measurement Considerations, and Intervention Implications. Sport and Exercise Psychology Research2016:373-88.

**Appendix 2:**

**Additional Trial Information**

**Data Storage**

The aquisition, storing and processing of all personal data will be conducted in accordance with GDPR, the Data Protection Act 2018, and University of Birmingham’s research governance frameworks. All participants will be pseudonymised by assigning a trial number upon initial consent being gained which will only be known to the chief and lead investigators and essential members of the ROH research team. The numerical code assigned to each participant will allow for identification of the participant and will only be used to establish contact to arrange and conduct outcome assessment, to invite to semi-structured interviews if needed, or to send a lay summary of results if of interest to the participant.

PROM data and accelerometers will be sent by participants to the research team at the ROH in the provided pre-addressed and stamped envelopes via post after their assessment sessions. PROMS, feasibility trial and semi-structured interview consent forms, performance-based outcome results, and accelerometers will be collected from the ROH by MW. Physiotherapists and ROH trial staff will complete focus their consent forms prior to the respective focus groups and MW will collect the physical copies of focus group consent forms. MW will scan all collected documents at the University of Birmingham. The semi-structured interviews and focus groups will be audio recorded using a digital recording device. If conducted via zoom, the video will not be recorded. The audio recording of the interview/focus groups and intervention sessions (for fidelity assessment) will contain no personal data when uploaded to the transcription website. All scanned documents, accelerometer data, pseudonymised interview/focus group transcripts, and intervention sessions will be uploaded and stored electronically on the secure REDcap system at the University of Birmingham by MW.

The original physical copies of all documents forms will be placed in a secure, locked filing cabinet in the office of the chief investigators (JD). The Accelerometer data and audio files on the digital recording device will be deleted after upload to REDcap. When not in use, the digital recording device used in semi-structured interviews and focus groups will be stored in the secure, locked filing cabinet in the office of the lead investigator. Therefore, only the chief (JD) and lead (MW) researchers will have access to participant data.

**Adverse Events**

Good Clinical Practice (GCP) defines two primary types of adverse events (Table 1)

**Table 1: Adverse Event Definitions**

| **Term** | **Definition** |
| --- | --- |
| **Adverse Event (AE)** | Any untoward medical occurrence in a participant to whom a medicinal product has been administered, including occurrences which are not necessarily caused by or related to that product. |
| **Serious Adverse Event (SAE)** | A serious adverse event is any untoward medical occurrence that:   - results in death - is life-threatening - requires inpatient hospitalisation or prolongation of existing hospitalisation - results in persistent or significant disability/incapacity - consists of a congenital anomaly or birth defect   Other ‘important medical events’ may also be considered serious if they jeopardise the participant or require an intervention to prevent one of the above consequences. |

No adverse events are expected beyond minor musculoskeletal pain which may arise from new and progressive PA /exercises prescribed at physiotherapy sessions. The physiotherapists will be mindful of any such events and the intervention will be modified as appropriate to reduce any symptoms. Any adverse events will be recorded at participant outcome assessments with research nurses and reported to the PI who will document the specifics of the event and duration. When an AE has occurred, which is suspected to be a potential SAE, the PI, lead and Chief investigator will discuss the causality and severity of the AE with the chair of the Trial Steering Committee. If the AE is confirmed as being a SAE, the CI will complete a SAE form, which will be scanned and emailed to the ROH, REC, and sponsor for review, where a local incident form will also be completed. For each SAE the following information will be collected:

1. Full details and case description of event.
2. Event duration.
3. Action taken.
4. Outcome.
5. Level of severity.
6. Causality

**Dissemination of findings**

The results are intended to be disseminated through publications in peer reviewed journals and presentations at appropriate physiotherapy and inter-disciplinary conferences. A lay summary of findings will be completed and sent to participants and interested members of the public (supported by the Patient and Public Involvement representative) and to the ROH, and a summary report will be produced for the sponsor (University of Birmingham) and research funders (Private Physiotherapy Educational Foundation and Musculoskeletal Association of Chartered Physiotherapists).

List of Abbreviations

HRA: Health Research Authority; IRAS: Integrated Research Application System; REDcap: Research Electronic Data capture; PPI: Patient and Public Involvement; MACP: Musculoskeletal Association of Chartered Physiotherapists; GCP: Good Clinical Practice. PALS: Patient advice and Liaison Service
